# Nocturnal Plant Bugs Use *cis*-Jasmone to Locate Inflorescences of an Araceae as Feeding and Mating Site

**DOI:** 10.1007/s10886-016-0688-9

**Published:** 2016-04-13

**Authors:** Florian Etl, Andreas Berger, Anton Weber, Jürg Schönenberger, Stefan Dötterl

**Affiliations:** Department of Botany and Biodiversity Research, University of Vienna, Rennweg 14, 1030 Vienna, Austria; Department of Ecology & Evolution, Plant Ecology, University of Salzburg, Hellbrunnerstr. 34, 5020 Salzburg, Austria

**Keywords:** Dynamic headspace, Floral scent, Florivory, Gas chromatography-mass spectrometry, Plant-animal interactions, (*Z*)-Jasmone, Heteroptera, Miridae

## Abstract

Inflorescences of Araceae pollinated by cyclocephaline scarab beetles are visited frequently by a wide array of other arthropods that exploit floral resources without taking part in pollination, including earwigs, flies, and true bugs. To date, nothing is known about the cues these insect visitors use to locate the inflorescences and whether or to what extent floral scents play a role. An aroid visited by large numbers of plant bugs (Miridae) in addition to cyclocephaline scarab beetle pollinators is the Neotropical species *Dieffenbachia aurantiaca*. We identified the plant bug species and investigated their behavior and arrival time on the inflorescences. To test the importance of olfactory cues in locating their host we conducted experiments with open and gauze-bagged inflorescences as well as natural scent samples of *D. aurantiaca*. Inflorescence scents were analyzed by gas chromatography linked to mass spectrometry (GC/MS), and the attractive potential of the main scent compound was determined by behavioral assays. Three species of *Neella*, the most common one being *N. floridula*, visited the inflorescences at nightfall, shortly after the beginning of scent emission, and showed feeding and copulation activity. Bagged inflorescences as well as natural scent samples attracted similar numbers of plant bugs as the non-bagged inflorescences, showing that olfactory cues are sufficient for them to locate their host. *Cis*-jasmone was the major component within the inflorescence scent bouquet. In two-choice field bioassays, this compound proved to be highly attractive to *Neella*, and thus obviously plays a key role in finding host plants.

## Introduction

With ca. 130 genera/3300 species, the Araceae are a speciose family of monocot angiosperms with a mostly tropical distribution and enormously varied pollination strategies. Pollination is achieved by many different kinds of insects (e.g., flies, beetles, bees), with large cyclocephaline scarab beetles (Coleoptera: Scarabaeidae: Dynastidae) playing a significant role in Neotropical genera, such as *Dieffenbachia*, *Philodendron*, and *Xanthosoma* (e.g., Garcia-Robledo et al. 2005; Gibernau et al. 1999; Young 1986). These scarab beetles arrive at night, attracted by intense floral scents (with emission supported by thermogenesis), use the inflorescences as feeding and mating sites, and effect pollination by their activities (e.g., Pereira et al. 2014). However, little attention has been paid to the fact that the inflorescences of many species are visited additionally by a great variety of other arthropods, including mites, earwigs, fruit and other flies, small beetles of various families, hymenopterans, and true bugs. They use the inflorescences as well as feeding/mating sites, but are mostly irrelevant for pollination (Garcia-Robledo et al. 2005; Gibernau et al. 1999; Valerio 1984; Young 1986). Among the non-pollinating visitors, plant bugs (Heteroptera: Miridae) are of particular interest. They are almost omnipresent in most cyclocephaline scarab beetle-pollinated inflorescences, and often occur in great numbers. In *D. nitidipetiolata* Croat & Grayum, they have been noted to arrive approximately at the same time as the nocturnal beetle pollinators (Young 1986). Thus, they seem to exploit the scent-driven communication channel between the plant and its beetle pollinators to find appropriate inflorescences as feeding and mating sites. This is supported by the finding that some diurnal mirid species have been shown to respond to plant volatiles (Koczor et al. 2012, see also references therein). However, in contrast to the increasing knowledge on the chemical compounds mediating the interaction between aroids and their cyclocephaline beetle pollinators (e.g., Dötterl et al. 2012; Pereira et al. 2014), nothing is known about the cues used by the plant bugs and other non-pollinating visitors to locate inflorescences of Araceae.

The present study deals with *Dieffenbachia aurantiaca* Engl. and its main non-pollinating visitors: species of the bug family Miridae. The following questions were addressed: (1) Which plant bug taxa are attracted? (2) At what time do they arrive? (3) How do they behave during anthesis? (4) Are olfactory cues sufficient for attracting them? (5) What is the chemical composition of the inflorescence scent? (6) Is the major scent component alone capable of attracting the plant bugs?

## Methods and Materials

### Plants, Study Time, and Study Site

*Dieffenbachia aurantiaca* is an endemic plant of the Golfo Dulce region of south-western Costa Rica and western Panama, and is pollinated by cyclocephaline scarab beetles (Etl et al. unpublished). It frequently grows in the understory of wet forests at swampy sites and river edges. Inflorescences consist of a flower-bearing spike, called the spadix, enclosed by a bract, called the spathe. The distal part of the spadix bears staminate (male) flowers, and the proximal part pistillate (female) ones. The latter part is surrounded by the somewhat inflated part of the spathe to form a pollination chamber. Anthesis starts with the pistillate phase (scented phase) followed by the staminate phase approximately 24 h later, i.e., the inflorescences are protogynous. The study was carried out during the dry seasons between February and April of three successive years (2013–2015) at the edge of the Piedras Blancas National Park, near the Tropical Research Station La Gamba, Costa Rica (8°42′46′′ N, 83°12′90′′ W).

### Responses and Behavior of Plant Bugs (Heteroptera: Miridae) to/on Bagged and Non-Bagged Inflorescences

We observed numerous inflorescences (*N* = 29) of *D. aurantiaca* in the field during the pistillate and the staminate phase from 17:30 (beginning of nightfall) until 22:00. To test the attractiveness of inflorescences when visual cues are excluded, we bagged 11 of these inflorescences with black (*N* = 6) or white (*N* = 5) fine-meshed nylon gauze bags. Arrival time, duration of stay, and behavior of the bugs as well as their numbers were noted. The number of attracted individuals to bagged and non-bagged pistillate phase inflorescences was statistically compared among the treatments using a *Kruskal-Wallis test*, Statistica 7.0 software package (StatSoft Inc., USA). Fourteen bug individuals from a few inflorescences were captured and preserved in 70 % alcohol for sexing and identification. As species could be distinguished easily by obvious color patterns, individuals of further inflorescences were identified in the field and not sexed.

### Scent Collection and Analyses

Using dynamic headspace methods (see Dötterl et al. 2012), we collected scents from six different inflorescences (5 individuals) of *D. aurantiaca* during the period of strong scent emission (pistillate phase; ca. 18:30; as determined by human nose and indicated by attraction of large numbers of mirid bugs during preliminary observations). Inflorescences were bagged with polyethylene oven bags (10 × 30 cm; Toppits, Germany), and scent was trapped for 2 min [either directly after bagging (*N* = 4) or 10 min after bagging (*N* = 2)] on adsorbent tubes (quartz glass tube: length 25 mm; inner diam 2 mm) filled with 1.5 mg each of Carbotrap B (mesh 20–40, Supelco, Germany) and Tenax TA (mesh 60–80; Supelco, Germany). For scent collection, a membrane pump (Gardner Denver, Germany) was used, and the flow was set at 200 ml/min. To obtain negative controls, we conducted the same procedure but with empty oven bags (*N* = 3).

Samples were analyzed by GC/MS (QP2010Ultra, Shimadzu Corporation, Japan) coupled to a thermal desorption unit (TD-20, Shimadzu, Japan) and equipped with a ZB-5 fused silica column (5 % phenyl polysiloxane; 60 m long, inner diam 0.25 mm, film thickness 0.25 μm, Phenomenex, USA). Samples were run at a column flow (carrier gas: helium) of 1.5 ml/min. GC oven temperature started at 40 °C, then increased by 6 °C per min to 250 °C, and held for 1 min. The MS interface worked at 260 °C, and the ion source at 200 °C. Mass spectra were taken at 70 eV (in EI mode) from *m*/*z* 30 to 350. The GC/MS data were processed using the GCMSolution Version 4.11 (Shimadzu Corporation, Japan). Compounds were identified tentatively by the NIST 11, Wiley 9, FFNSC 2, Essential Oils and Adams 2007 mass spectral data bases, and were confirmed by comparison of mass spectra and retention times with those of authentic standards (*cis*-jasmone: Sigma Aldrich, 85 %; methyl benzoate: Sigma Aldrich, 98 %; methyl salicylate: Sigma Aldrich, 99 %; *trans*-4,8-dimethylnona-1,3,7-triene: available in the stock collection of SD, 95 %). To determine the amount of scent emitted from an inflorescence (*N* = 4; samples collected without accumulation time, see above), known amounts of monoterpenes, aliphatics, and aromatics were injected into the GC/MS system; mean peak areas of these compounds were used to determine the total amount of scent (see Dötterl et al. 2012).

To obtain a natural scent sample for bioassays (see below), floral scent was collected as described above, but with a bigger adsorbent tube (Pasteur pipette). The tube was filled with 20 mg each of Carbotrap B and Tenax TA (see above). One inflorescence was sampled during the pistillate phase for 3.5 h (18:30–22:00), and trapped scent was eluted with 1 ml acetone (p.a., Merck, Germany) into a glass vial and stored at minus 20 °C. This sample was used for all bioassays.

### Field Bioassays

We tested either the natural scent sample (offered in the glass vial, *N* = 5) or synthetic *cis*-jasmone (Sigma Aldrich, USA >85 %) (1 ml in a glass vial, *N* = 2; or 60 μl on a filter paper, *N* = 3) in a series of two-choice bioassays against negative controls (same amount of acetone or an empty filter paper). Bioassays started at 17:30 and lasted until 21:00. Arriving plant bugs were counted every 30 min, and the highest number per experiment was taken for statistical analyses. At the end of each experiment, attracted plant bugs were captured to prevent counting a single individual more than once (pseudo replication). The number of plant bugs attracted by lures and controls was statistically compared using an *exact binomial tests of goodness-of-fit* (http://www.biostathandbook.com/exactbin.xls). We identified >100 individuals attracted during the different assays to species level. Since the species composition consisted mainly of one species [*Neella floridula* (Distant, 1883), see below], and no bugs of other genera were attracted, we did not discriminate among species of *Neella* in the statistical tests.

## Results and Discussion

**Fig. 1:**
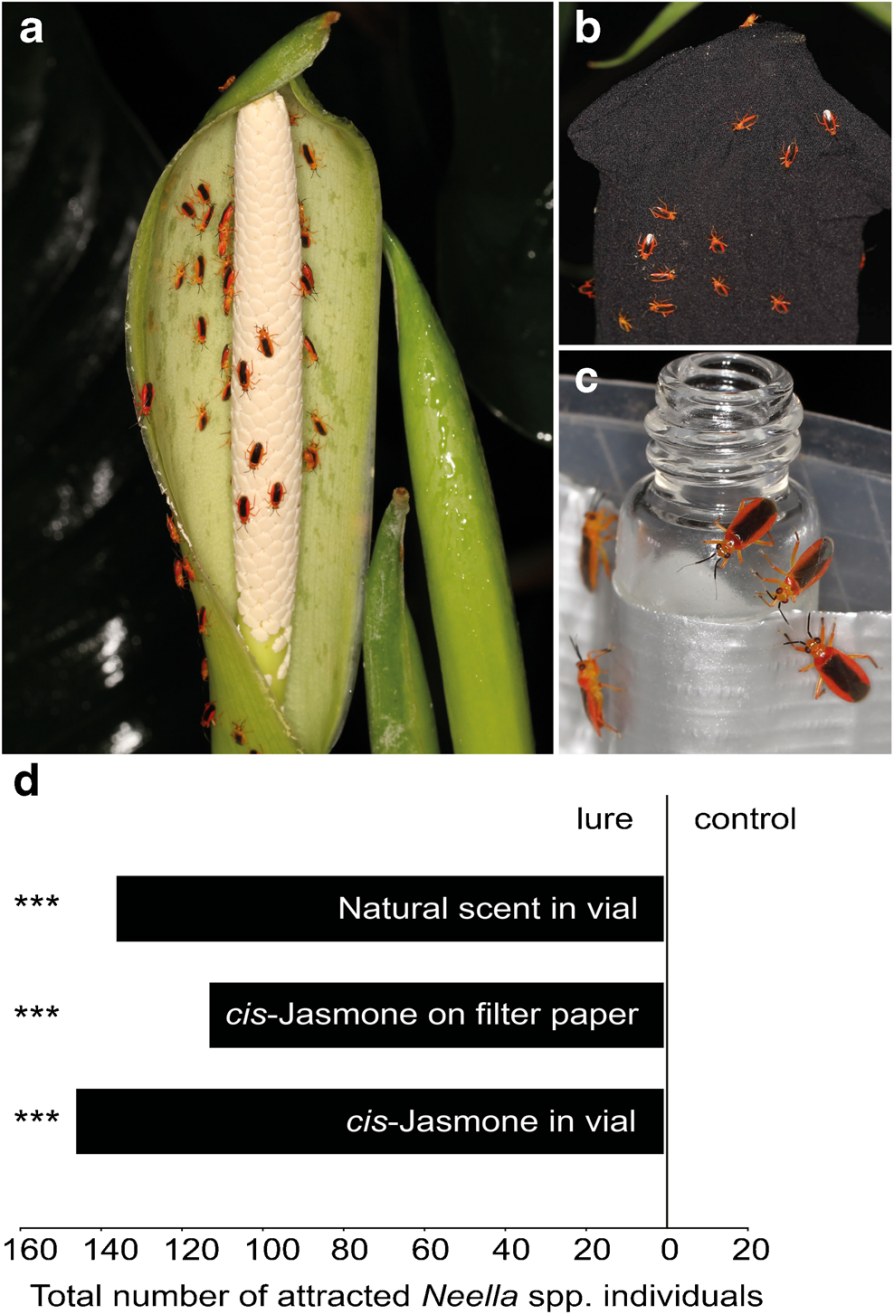
Numerous individuals of *Neella floridula* on **a** the spathe and the staminate (male) part of a spadix of *Dieffenbachia aurantiaca*, **b** a bagged inflorescence, and **c** a glass vial containing a natural scent sample. **d** Total number of attracted *Neella* spp. individuals in a series of two-choice bioassays with a natural scent sample (1 ml in glass vial; 5 assays pooled) and synthetic *cis*-jasmone applied on filter paper (60 μl; 3 assays pooled) or offered in a glass vial (1 ml; 2 assays pooled), all tested against negative controls. *Exact binomial tests* (lure vs. control): ***: *P* ≤ 0.001

During all standardized observations, there was only one plant bug species visiting the inflorescences of *Dieffenbachia aurantiaca*: *Neella floridula* (Heteroptera: Miridae: Bryocorinae). The 14 sexed individuals had a sex ratio of 6:1 (females: males). During occasional observations, we additionally observed rare visitations by *N. bicolor* (Hsiao, 1946) and *N.* cf. *carvalhoi* (Hsiao, 1946). Bugs started to arrive at inflorescences after the beginning of scent emission at around 18:30, during the pistillate phase (Fig. 1a) and stayed there for 24 h, until the end of anthesis. Immediately after arrival, they inserted their proboscides (piercing sucking mouthparts) into the staminate portion of the spadix, between the densely aggregated male flowers, likely to feed on pollen (Wheeler 2001). Only rarely did they feed on the spathe. When numbers of *Neella* bugs were high, some individuals also settled on nearby leaves. A small proportion of individuals copulated, making *D. aurantiaca* not only a feeding but also a mating site for *Neella* spp. The bugs rarely entered the pollination chamber, and if, they only sat on the inner wall of the spathe, not touching the female receptive organs. Consequently, these florivores were excluded as pollinators (see also Gibernau et al. 1999; Valerio 1984; Young 1986).

Non-bagged inflorescences of *D. aurantiaca* (Fig. 1b; Mean ± SE: 27 ± 6; Min-Max: 5–120) attracted similar numbers of *Neella* bugs as inflorescences with white (20 ± 7; 6–50) and black (Fig. 1a; 20 ± 4; 4–32) gauze bags (H_2, *N* = 29_ = 0.51; *P* = 0.77). This suggested that olfactory cues alone are sufficient for the bugs to locate their host. However, as we could not exclude the effect of thermogenesis on the attraction of the bugs, the importance of olfactory cues apart from heat emission was confirmed by bioassays using natural scent samples. These samples also attracted a high number of *Neella* bugs (Mean ± SE: 27 ± 11; Min-Max =1–70) in field bioassays (Fig. 1c). In total, 136 individuals were attracted to natural scent samples and no bug responded to negative controls (Fig. 1d).

The total amount of scent emitted per inflorescence of *D. aurantiaca* was high (Mean ± SE: 39 μg/h ± 25; Min-Max: 4–126), but somewhat lower than in other cyclocephaline beetle-pollinated plants, such as *Philodendron* aff. *bipinnatifidum* (Dötterl et al. 2012). The scent bouquet of *D. aurantiaca* contained nine volatile compounds (Table 1) with *cis*-jasmone being the major component (49 %), followed by an unknown compound (Kovats retention index: 1317; 30 %) and methyl salicylate (18 %).

**Table 1:** Relative amounts (mean ± SD) of inflorescence scent compounds of *Dieffenbachia aurantiaca* and their occurrence in the 6 samples analyzed. Values >10 % are printed in bold

	Kovats retention index	Occurrence	Mean % (± SD)
Aromatics			
Methyl benzoate*	1101	6	0.24 ± 0.19
Methyl salicylate*	1207	6	**18.21** ± 5.78
Terpenoid			
*trans*-4,8-Dimethylnona-1,3,7-triene*	1117	3	0.08 ± 0.12
Miscellaneous			
*trans*-Jasmone*	1399	1	< 0.01
*cis*-Jasmone*	1413	6	**49.36** ± **11.2**
Unidentified compounds			
*m*/*z*: 41.91.94.105.133	1301	5	0.24 ± 0.27
*m*/*z*: 41.91.94.105.133	1317	6	**29.64** ± 5.4
*m*/*z*: 39.79.93.135.150	1327	5	0.09 ± 0.09
*m*/*z*: 41.53.81.108.110	1390	5	2.13 ± 2.36

*Authentic standards were available

Vials with 1 ml of pure *cis*-jasmone attracted a total number of 146 individuals (Mean ± SE: 73 ± 20, Min-Max =45–101) of *Neella* bugs, and lures containing a filter paper with 60 μl of pure *cis*-jasmone attracted a total of 113 individuals (38 ± 2, 32–41), while controls did not attract any bugs (Fig. 1d). Species composition of *Neella* attracted to *cis*-jasmone lures was similar as on inflorescences of *D. aurantiaca*. Out of 100 individuals attracted to *cis*-jasmone, 84 were *N. floridula* (sex ratio females: males =1:2) and 16 were *N. bicolor* (sex ratio females: males =1:4). The sex ratio of *N. floridula* attracted to *cis*-jasmone differed from the sex ratio of the bugs collected on inflorescences (see above). However, it is unclear whether this difference is due to stochastic effects (only a small number of bugs collected on inflorescences was sexed) or that *cis*-jasmone attracts the sexes differently when compared to an inflorescence. Based on measurements by Dötterl et al. (2012), the amount of *cis*-jasmone (60 μl) applied on filter papers in the present study resulted in 3 μg scent emitted per hour, which lies within the range of *cis*-jasmone emitted by *D. aurantiaca* (Mean ± SE: 21 μg/h ± 15; Min-Max =2–72 μg/h). The amount of scent released by the glass vials was not measured but was perceived as being weaker for the human nose than that perceived from filter paper. However, scent was emitted constantly for a longer period due to the small opening of the vials. Altogether, our results show that the major component in the inflorescence scent of *D. aurantiaca* attracts similar numbers of *Neella* spp. as the natural scent, indicating that *cis*-jasmone is a strong olfactorial cue for *Neella* spp. in search of food and mating sites. Our experimental setup did not exclude the possibility that bugs attracted by *cis*-jasmone contributed to the attraction of congeners. However, we believe that attraction by pheromones was of minor importance or did not occur, as *Neella* bugs kept in a fine-meshed cage did not attract other bugs in the absence of plants/scents (Etl, unpublished data).

*Cis*-jasmone is widespread among floral scents (Knudsen et al. 2006) and also occurs in several other aroids pollinated by cyclocephaline scarab beetles (Pereira et al. 2014). Some of these aroids (*Montrichardia arborescens* Schott and *Philodendron bipinnatifidum* Schott ex Endl.) also emit *cis*-jasmone as their major scent component, while it is a minor component in other species (e.g., *Caladium bicolor* (Aiton) Vent., *P*. aff. *bipinnatifidum*; Pereira et al. 2014). At least the two aforementioned *Philodendron* species also are visited by a *Neella* species (Gerhard Gottsberger, Ulm University, pers. Comm.), and this so far unidentified species might also use *cis*-jasmone as a cue to find host plants. In *P*. aff. *bipinnatifidum*, *cis*-jasmone was shown to increase the attractiveness of other more specific compounds for cyclocephaline beetle pollinators, but it failed to attract beetles when tested alone (Dötterl et al. 2012). During the present study, we attracted one individual of a cyclocephaline beetle pollinator with synthetic *cis*-jasmone. More experiments are necessary to test whether this compound is not only a strong attractant for plant bugs but also a reliable pollinator attractant in *Dieffenbachia aurantiaca*.

Overall, our study shows for the first time that nocturnal florivorous Miridae use inflorescence scent, specifically *cis*-jasmone, a compound also known as an attractant for florivorous thrips (El-Sayed et al. 2009) and as a plant defense volatile (Birkett et al. 2000), to locate their feeding and mating site. Preliminary data suggest that this compound also is relevant for the attraction of the cyclocephaline scarab beetle pollinators. This supports the hypothesis that floral scents are attractive not only to pollinators but also to florivores and, therefore, may be under counter-acting selective pressures (Schiestl 2015).
